# Cost Effectiveness and Budget Impact Analyses of Influenza Vaccination for Prisoners in Thailand: An Application of System Dynamic Modelling

**DOI:** 10.3390/ijerph17041247

**Published:** 2020-02-14

**Authors:** Rapeepong Suphanchaimat, Pawinee Doung-ngern, Kritchavat Ploddi, Suthanun Suthachana, Mathudara Phaiyarom, Kumaree Pachanee, Walairat Chaifoo, Sopon Iamsirithaworn

**Affiliations:** 1 Division of Epidemiology, Department of Disease Control, Ministry of Public Health, Nonthaburi 11000, Thailanddr.kritchavat@gmail.com (K.P.); suthachana@gmail.com (S.S.); walaich@yahoo.com (W.C.); 2 International Health Policy Program (IHPP), Ministry of Public Health, Nonthaburi 11000, Thailand; mathudara@ihpp.thaigov.net (M.P.); kumaree@ihpp.thaigov.net (K.P.); 3 Division of Communicable Diseases, Department of Disease Control, Ministry of Public Health, Nonthaburi 11000, Thailand; iamsiri@gmail.com

**Keywords:** influenza, prisoners, vaccine, cost effectiveness, budget impact, Thailand

## Abstract

Influenza outbreaks in Thai prisons were increasing in number every year and to address this, the Thai Ministry of Public Health (MOPH) initiated a policy to promote vaccination for prisoners. The objective of this study was to assess the cost effectiveness and budget impact of the influenza vaccination policy for prisoners in Thailand. The study obtained data from the Division of Epidemiology, Department of Disease Control (DDC), MOPH. Deterministic system dynamic modelling was exercised to estimate the financial implication of the vaccination programme in comparison with routine outbreak control. The incremental cost-effectiveness ratio (ICER) was calculated via a DDC perspective. The reproductive number was estimated at 1.4. A total of 143 prisons across the country (375,763 prisoners) were analysed. In non-vaccination circumstances, the total healthcare cost amounted to 174.8 million Baht (US$ 5.6 million). Should all prisoners be vaccinated, the total healthcare cost would reduce to 90.9 million Baht (US$ 2.9 million), and 46.8 million Baht (US$ 1.5 million) of this is related to the vaccination. The ICER of vaccination (compared with routine outbreak control) varied between 39,738.0 to 61,688.3 Baht per disability-adjusted life year (DALY) averted (US$ 1281.9–1989.9). Should the vaccination cover 30% of the prisoners, the ICER would be equal to 46,866.8 Baht (US$ 1511.8) per DALY averted with the budget burden amounted to Baht (US$ 4.8 million). The vaccination programme would become more cost-effective if the routine outbreak control was intensified. In summary, the vaccination programme was a cost-effective measure to halt influenza outbreak amongst prisoners. Further primary studies that aim to assess the actual impact of the programme are recommended.

## 1. Introduction

Seasonal Influenza has been long recognized as a vital cause of morbidity and mortality in humans for years [1]. In 2017 the World Health Organization (WHO) estimated that seasonal influenza was associated with all-cause mortality in about 250,000–500,000 deaths annually [2]. Those who are at great risk of serious complications and mortality are mostly vulnerable populations, including people with immuno-compromised host, young children, and the elderly [3,4]. Thailand is amongst the countries where seasonal influenza is of vital public health concern. Since 2004, the Thai Ministry of Public Health (MOPH) has set explicit goals to reduce the burden of influenza in Thailand, both for the advancement of public health and the maintenance of national security [5]. 

Vaccination is proven to be one of the most effective measures to prevent influenza transmission and reduce morbidity and mortality, particularly in groups at risk of complications, including young children and the elderly. The selection of strains to include in annual influenza vaccines is based on global surveillance of circulating influenza viruses; then at the beginning of each year, WHO announces the viral strains that are commonly found in the northern and southern hemispheres. Predictions are made months prior the advent of ‘flu season’ in order to accommodate all the steps of vaccine production, including the generation of seed viruses, amplification, inactivation, purification, and dispensing [6,7]. A fair amount of literature suggests varying degrees of vaccine effectiveness in preventing seasonal influenza. Ohmit et al. from the US reported that vaccination was able to prevent community-acquired influenza by 31% [8]. A two-year prospective cohort study amongst older adults in Thailand showed that, given a well antigenic match between the dominant circulating virus and vaccine strain, vaccination could help reduce the number of patients by almost 50% [9]. However, there are always challenges in the vaccination. These include the lengthy time of vaccine production, the fall in vaccine-specific antibodies over time, and the failure of strain-specific immunity to protect against drifted seasonal influenza viruses or from antigenically novel viruses (like in the case of pandemic 2009 H1N1 influenza) [7,10].

In Thailand, the seasonal vaccination policy has evolved over time. The important milestone occurred in 2008 when the National Health Security Office (NHSO), the governing body of the national insurance scheme, included influenza vaccine in the insurance’s benefit package. The target populations comprise seven groups of high-risk people: (i) elderly aged over 65 years, (ii) persons with chronic diseases or immuno-compromised conditions, (iii) obese persons, (iv) persons with neurological impairment, (v) Thalassemic patients, (vi) children aged from six months till two years, and (vii) pregnant women with gestational age over 34 weeks [11]. Vaccines purchased were inactivated trivalent vaccines and were allocated to each province according to the estimated number of target populations.

It should be noted that prisoners are not amongst the seven high-risk groups specified above despite the fact that influenza outbreaks occurred in prisons from time to time. Evidence from the Division of Epidemiology (DOE), Department of Disease Control (DDC), MOPH, revealed that the toll of influenza outbreaks in Thai prisons showed a rising trend, from 17 events in 2017 to 28 events in 2019. Each outbreak caused 81–105 infected persons on average. Influenza-A H1N1 was the dominant viral strain during 2017–2018, yet in 2019 influenza-B became the dominant strain. Note that, as of 2019, there were 143 prisons in Thailand. Of these 143 prisons, 69 had suffered from an outbreak at least once during the last three years; and 38 of these 69 prisoners had experienced an outbreak at least twice, Table 1.

Though the death tolls from the outbreaks were not substantial, all of these events meant a huge cost of care and actions for outbreak control. In the status quo, when the outbreak takes place or when a cluster of infected people is detected, the DOE is obliged to deploy the rapid response team to investigate and control the event. Such a function is routinely operated by the Office of Disease Prevention and Control (ODPC), which is situated in twelve major provinces throughout Thailand. The ODPC can request the investigation team directly from the DOE if necessary (such as during an outbreak with a huge volume of infected cases or an event that necessitates special laboratory testing that is beyond the capacity of the local response teams).

The growing trend of influenza outbreaks in prisons led to a policy dialogue amongst various stakeholders, including representatives from the Thai DDC, the NHSO and the Department of Corrections (DOC), Ministry of Justice (MOJ) regarding the benefit of a vaccination policy for prisoners in order to bring down the trend. The vaccination initiative requires a meticulous consideration concerning the potential cost of investment in the vaccines and potential savings on treatment and outbreak-control expenses. The Thai-DDC therefore commissioned a working group comprising researchers from the International Health Policy Program (IHPP) and the Thai-DDC, MOPH, to conduct a rapid assessment on the cost effectiveness of the influenza vaccination policy for prisoners in Thailand as well as the potential budget impact.

## 2. Methods

### 2.1. Study Design and Data Sources

A quantitative secondary data analysis was employed. Data were obtained various sources, including the Thai-DDC, the NHSO, and the IHPP. An additional review was conducted on the website of the Centers for Disease Control and Prevention of the US (US-CDC), relevant international journals published in the MEDLINE database and domestic journals published in the Thai-DDC’s archive. The review focused on articles published after 2000. As this work involved a rapid assessment for the Thai-DDC, no specific inclusion and exclusion criteria were applied. However, some key search terms, like ‘influenza’, ‘vaccine’, and ‘prison’, were utilized. The primary aim of the review was to identify key parameters that were essential for the calculation. During the review process, investigators attempted to identify literature that focused on influenza in Thai domestic sources first. If such literature was not available, foreign literature was searched instead. Literature that focused on prisoners was preferable; but if unobtainable, studies on the general population were used as an alternative. Details of the parameters retrieved from the review are shown in the later subsection, *‘Model validation and parameter list’*. 

### 2.2. Model Framework

The compartment susceptible-infected-recovered (SIR) model and system dynamic (SD) model were applied to and adapted for this study. The SIR model categorised the concerned population into three subgroups (compartments): the susceptible, the infected and the recovered [13]. The susceptible people would become infected people once in contact with the infected cases. The reproductive number (R0) was considered the number of new cases generated as a result of the contact with a case, amongst totally susceptible population, throughout the infectious period [14]. 

The majority of infected cases had mild symptoms, which required only simple outpatient treatment. Some patients experienced a severe clinical course which necessitated inpatient care. Most patients soon recovered from the disease, and very few of them died. Vaccination was proven to be one of the most effective measures to prevent the move from susceptible compartment to infected compartment (as some of the vaccinees were immune, with varying degrees according to the vaccine efficacy) [15]. Moreover, the influenza vaccine was capable of shortening the duration of symptoms and reducing the severity of disease [16]. This meant, amongst the vaccinees, the transition from infected compartment to recovered compartment would be faster than the non-vaccinees.

The SD model captured the transition from one group to another, taking into account factors that potentially influence the transition (in terms of both probability and rate) and multiple interactions amongst such factors, including delivery systems, resource availability and contextual policies [17]. That is, the effectiveness of the vaccination policy could not be solely estimated by the clinical efficiency of the vaccine per se, and other measures are normally undertaken to tackle the outbreak in tandem.

One of the most common measures for terminating the outbreak was the deployment of a rapid response team by the DOE; this was routinely activated when the accumulated cases in a prison amounted to 30–50 cases. The main activities of the team in the field included (but were not limited to) the provision of health education to prisoners and wardens, prescription of Oseltamivir to suspected cases, and specimen collection for further laboratory testing.

The primary output of the models was the calculation of the volume of prisoners in each compartment (susceptible, infected and recovered) at a particular point in time. The time span of the calculation was one year. Then the cost of vaccination, cost of care and quality of life (in terms of disability adjusted life years [DALY]) were estimated by multiplying the number of cases by the outcome of interest—for instance the overall DALYs = [(number of infected prisoners x DALYs for each infected case) + (number of dead prisoners x DALYs for each death)].

The deterministic analysis was performed from the Thai-DDC’s perspective. The final outcome of the model was the incremental cost effectiveness ratio (ICER), which was calculated by dividing the total cost (vaccination cost and outbreak investigation cost combined) by the volume of DALY averted by each vaccination policy (ICER = $\frac{cost_{policy}-cost_{no\;policy}}{DALYs_{no\;policy}-DALYs_{policy}}$). The cost of treatment for infected prisoners was not directly used to estimate ICER. The treatment cost was normally absorbed into the routine services of public facilities (including healthcare centres in prisons). In other words, it was not an additional investment of the policy.

**Figure 1:**
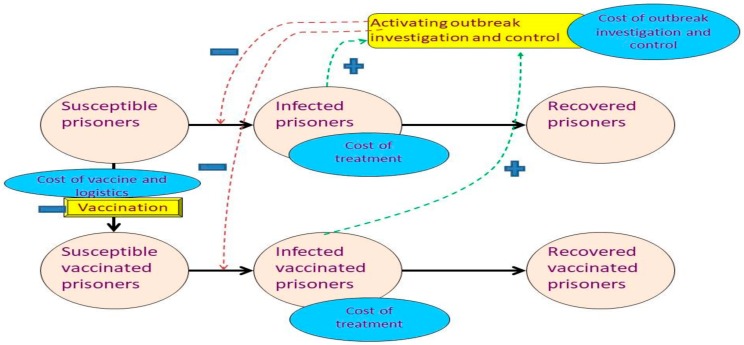
Brief framework of the model. Note: (+) = activating or accelerating the transition; (−) = deactivating or decelerating the transition

The model of interest relied on some key assumptions. Firstly, it is presumed that mass vaccination for prisoners could be performed within one day without any logistic delay. Secondly, there was no in- and out-migration to and from the prisons. However, a sensitivity analysis was performed to assess the change of cost-effectiveness if a dynamic population was assumed (more details in the ‘Sensitivity analysis’ subsection). Thirdly, a contact between a case and each (susceptible) person happened at random. Fourthly, all infected prisoners were treated. Those with mild symptoms were treated as outpatients whereas severe cases were treated as inpatients. The cost of Oseltamivir prescribed to mitigate the outbreak and the cost of outbreak investigation and control was totally borne by the Thai-DDC whereas the cost used for treatment was shouldered by the facilities. Fifthly, as the economic cost for each parameter was difficult to specify, monetary charge was used instead based on the hypothesis that the monetary charge approximately reflected the economic cost. Sixthly, the treatment cost for the side effects of influenza vaccination was ignorable. Lastly, no prisoners were assumed to have natural immunity against seasonal influenza at the beginning of a year. All of the above information explains how the model was constructed. Figure 1 demonstrates the simplified model framework. The full model framework is shown in Supplementary File 1. Details of the parameters used in the model and the vaccination programmes are elaborated later.

### 2.3. Model Validation and Parameter List

As briefly mentioned earlier, most parameters were retrieved from academic articles. Some parameters were obtained from the routinely collected records of the Thai-DDC, for instance the mean cost of laboratory investigation per outbreak (including rapid test for influenza and polymerase chain reaction [PCR] of nasopharyngeal swab). Some parameters relied on experts’ opinions and were estimated during model calibration. These parameters included average R0 of influenza in Thai prisons and the detection rate of infected cases in prisons. This is because in many circumstances during outbreak investigation, the rapid response team discovered that the actual cases in the field far outnumbered the reported cases. In this regard, a series of consultative meetings with epidemiological experts and physicians in the Thai-DDC were arranged. Three rounds of meetings were held. Each round contained five to six participants and lasted about 45 minutes. The participants were asked to brainstorm and comment on the model (in terms of completeness, correctness and estimation of key parameters). The model was calibrated by running the model with all acquired parameters. The model outputs (such as number of outbreak investigations and number of deaths) were calibrated against the historical records of influenza outbreaks in prisons (as shown in Table 1). The estimated parameters were readjusted until the model outputs best matched the historical records. An example of the results from model calibration was, given a 90% detection rate for outbreak investigation, the total number of events being investigated would amount to 172, far larger than the actual number of investigated events. However, when a 45% detection rate was used, the total volume of events being investigated was approximately 28. These numbers also coincided with the views of the experts who had prior experience in field investigation. Vensim ^®^ PLE7.2 software was used to run the model. Table 2 displays the values of key parameters. Table 3 demonstrates the features of the essential formula used in the model.

### 2.4. Model Choices

This study assumed four main vaccination scenarios: (i) no vaccination at all—‘Routine’, (ii) 10% vaccination—‘Vac10’, (iii) 30% vaccination—‘Vac30’, and (iv) 100% vaccination—‘Vac100’. The 10% figure was selected according to the data of the Thai-DDC, which showed that about 10% of prisoners were suffering from chronic non-communicable diseases (NCDs). Consequently, this group was likely to be the priority for the vaccination programme. The 30% figure was considered because, in theory, to acquire herd immunity, the vaccine coverage in a population must achieve a certain level, which could be estimated from R0 (herd immunity threshold = 1−1/R0, given perfect vaccine efficacy). With R0 = 1.40, the vaccine coverage to reach herd immunity threshold was approximately 30%. The 100% figure represented a setting that acquired full coverage.

Moreover, the experts of the Thai-DDC recommended that the analysis should account for the change in sensitivity of the activation of a rapid response team to control the outbreak. Thus the researchers assessed the programme’s cost effectiveness and potential budget impact in a situation when the sensitivity for the deployment of a rapid response team was doubled (in other words, the threshold of cases that was used for triggering the team lowered by half—from 30–50 to 15–25). This scenario was called ‘Extra’. In summary, there were eight scenarios of interest, Table 4. 

### 2.5. Sensitivity Analysis

Sensitivity analysis was performed as complementary to the base analysis. While the base analysis relied on close population assumption, the sensitivity analysis accounted for population dynamics. The DOC internal database showed, on average, a slight declining trend in the number of prisoners: about 2.2 prisoners released per day per prison in contrast to the daily admission rate of 2.1 prisoners. With this information, it is vital to assess to what extent the outflow and inflow of prisoners caused the change in the programme’s cost effectiveness.

## 3. Results

Overall, it is found that in Routine and Routinevac10 scenarios, the number of infected prisoners (both mild and severe infections) reached approximately 13,000 people by day 60; the greatest peak amongst all interested scenarios. The amount of infected prisoners markedly declined when at least 30% vaccination coverage was achieved. For instance, in the Routinevac30 scenario, the infected population amounted to only 8000 by day 70, which is about a 40% decrease compared with a no-vaccination scenario. 

In the setting where outbreak control was more sensitive, a varying degree of vaccination coverage (Extra, Extravac10, and Extravac30) did not exhibit distinct difference in terms of the peak volume of infected prisoners. The obvious difference was noticed in the form of disease propagation rate. In Extravac30 scenario, the outbreak propagated more slowly and subsided faster than in Extra and Extravac10 scenarios. It should be noted that all scenarios faced inconsiderable amount of death cases (< 2 cases).

**Figure 2:**
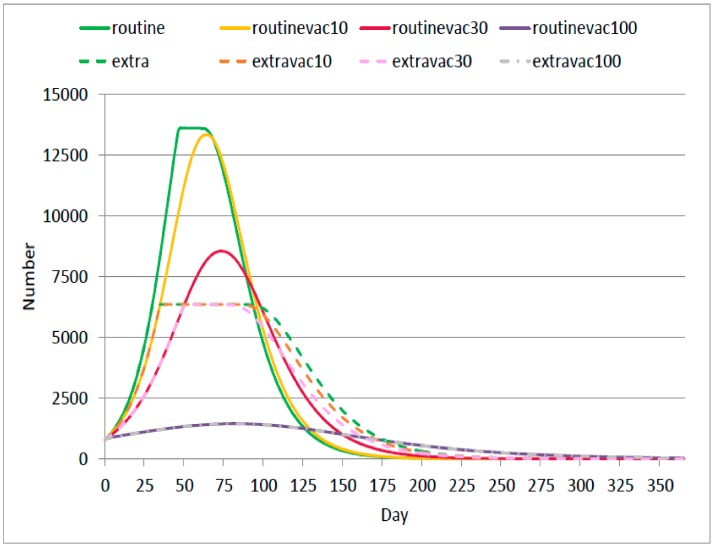
Number of infected cases at any point of time in a year in different scenarios.

In the full coverage scenario, infected cases reached a peak at about 1500. The Extravac100 scenario yielded the same result as the Routine100 scenario. This meant the number of cases at any point in time did not reach the outbreak investigation threshold when all prisoners were vaccinated, see Figure 2.

Routine and Routinevac10 scenarios incurred an accumulated (total) cost of about 170 million Baht (US$ 5.5 million), the greatest value in relation to other candidate scenarios. Routinevac30 and Extravac30 scenarios incurred a total cost of approximately 130 and 120 million Baht respectively (US$ 4.2 million and 3.9 US$ million). 

Corresponding to the analysis on patient volume, Routinevac100 and Extravac100 scenarios faced the same total cost of about 90 million Baht (US$ 3.9 million), the lowest amongst all scenarios. 

**Figure 3:**
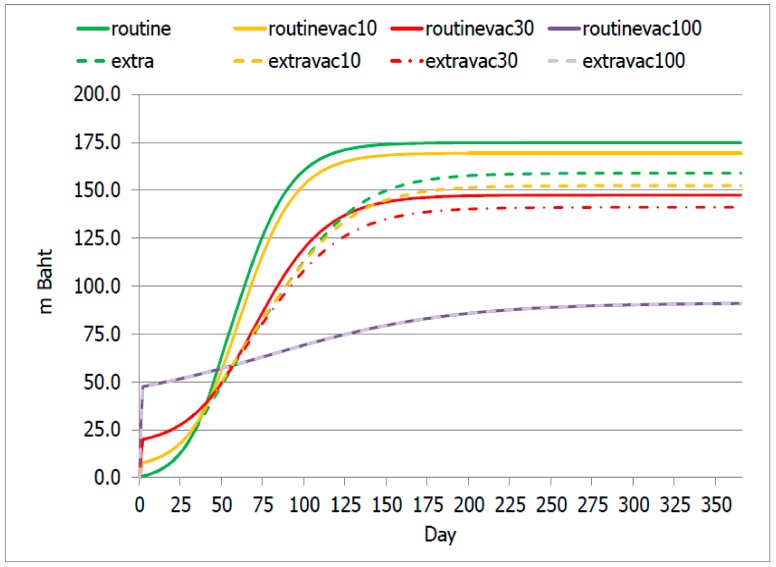
Accumulation of total cost over time.

During the first 60 days, the total cost in Routinevac100 and Extravac100 scenarios exceeded other scenarios, but after day 60, the full coverage scenarios led to a lower total cost relative to other candidate scenarios, Figure 3.

Table 5 presents the volume of cases, DALYs and all related costs accumulated throughout a year in each scenario. The routine scenario showed not only the largest DALYs but also the largest volume of monetary loss. The more prisoners were vaccinated, the lesser the total cost incurred (despite the increased investment in vaccination-related cost). DALYs appeared to have a negative relationship with the vaccination coverage. Routinevac100 and Extravac100 scenarios yielded the same outputs in terms of all costs combined, number of cases and DALYs.

**Figure 4:**
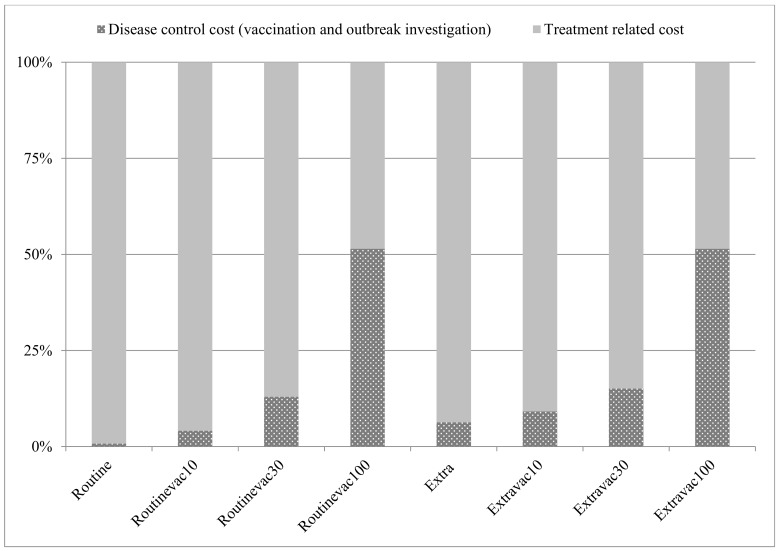
Share of disease control cost and treatment-related cost in each scenario.

In the full vaccination setting, the disease control cost (vaccination cost plus outbreak investigation cost) was almost on a par with the treatment-related cost; the greater the vaccination coverage, the smaller the share of treatment cost, Figure 4.

Given the analysis was confined to disease control cost, with reference to the Routine scenario, the full vaccination coverage appeared to be the most cost-effective investment amongst all interested scenarios, as evidenced by the smallest ICER at about 39,738.0 Baht per DALY averted (US$ 1281.9). In contrast, the Routinevac10 scenario contributed to the greatest ICER at approximately 61,688.3 Baht per DALY averted (US$ 1989.9). The Extra scenario was found to be the second cost-effective programme. The Extravac10 and Extravac30 scenarios exhibited a very small difference in ICER, Table 6.

**Figure 5:**
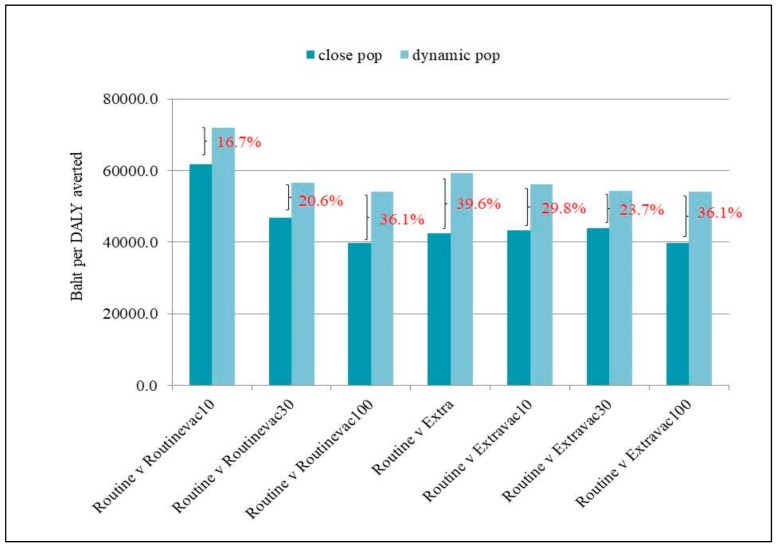
ICER in dynamic population assumption relative to close population assumption.

Similar to the base analysis, the full vaccination programme was shown to be the most cost-effective approach, compared with the other scenarios. In terms of self-comparison, the dynamic population assumption, as shown in the sensitivity analysis, presented less cost-effective outputs (larger ICER) relative to close population assumption by approximately 16.7–39.6, Figure 5.

## 4. Discussion

### 4.1. Result Discussion

The vaccination programme for prisoners clearly showed a cost-effective outcome. Compared with routine outbreak control, the vaccination policy helped reduce the number of infected cases and overall cost for treatment and outbreak control. This evidence clearly suggests that the investment in the vaccination policy for prisoners was worthwhile. Findings from international literature also point in the same direction. For instance, Guillermo-Sequera et al. flagged that access to vaccination amongst prisoners brought about a positive impact not only on the target population, but also in wider communities [28]. 

For the Thai setting, the existing national influenza vaccine programme targets vulnerable populations only. Following this idea, logic says that only 10% of prisoners would be vaccinated. The findings above (Figure 2 and Figure 3) obviously demonstrate that the benefit of the 10% vaccination coverage is not remarkably different from non-vaccination at all. One of the possible explanations for this phenomenon is, in congested space like prisoners, the contagiousness of disease was found to have great potential [20,29]. Hence, vaccination coverage that did not lead to herd immunity likely fails to protect the health of the whole community. A meaningful reduction in the number of cases was found when at least 30% coverage was reached. Of note is that, in practice, the degree of needed coverage should always account for vaccine efficacy as well.

Some may argue that implementing standards for infection control in prisons might suffice, with no need to implement a vaccination programme. Yet, in reality, due to many reasons (such as security concerns and budget constraint), most prisons did not meet the required hygienic standards and preventing the contact between susceptible case and infected case is not always feasible. Maruschak et al. [30] reported that, in the US, although standards did exist for infection control programmes in jails, only 350 jails (less than 10% of all jails nationwide) were accredited by official accrediting bodies with health standards. The results from this study also corroborated that vaccination programmes combined with stringent outbreak surveillance contributed to a favourable result compared with a vaccination policy alone. That is, with a population detained in a confined space, prisons provided a paradigmatic opportunity for vaccination interventions [31].

Notably, the Extra scenario represented the second most cost-effective programme after the full vaccination scenario, with the cost (shouldered by the Thai-DDC) four to five times lower than the cost of the full vaccination programme. This alludes to the fact that, even without vaccination, a stringent surveillance system on influenza still has significant health benefits, with good value for money [32]. Accordingly, the influenza surveillance system in prisons needs to be set up with mutual collaboration between healthcare providers and jail officers. Moreover, better infection control inside prisons would have a positive health impact on surrounding communities; and vice versa [28,29,30]. In this respect, rigorous infection control should be established as a default in everyday practice, not only when the outbreak takes place. 

Yet given the fluidity of prisons’ inmate populations, implementing infection control policies in jails may not be as easy as it sounds. The sensitivity analysis clearly demonstrated that in a dynamic population, all control programmes, regardless of the percentage of vaccination coverage, became less cost effective relative to close population settings (Figure 5). The short turnover times of prisoners pose substantial challenges in implementing infection control practices and adding procedures to control infection usually means the increase in roles, responsibilities, and administrative burden of the jail officers [30].

From the Thai-DDC’s perspective, the influenza vaccination policy comes with a trade-off to increase investment. The more prisoners are vaccinated, the more investment is required; and the authority has to set aside additional budget for vaccination at the beginning of the year. Hence, the issue rests with the budget capacity of the Thai-DDC. In an actual setting, the Thai-DDC may consider prioritising the prisons at risk based on historical data. Of the 143 prisons nationwide, 38 had experienced a seasonal influenza outbreak for at least two consecutive years in the last three years. These prisons likely faced R0 greater than 1.40. Some experts in the Thai-DDC estimated that R0 of these ‘high-risk’ would probably climb up to 3, and it is valid to provide 100% vaccine coverage to these prisons, while the rest of the prisons may warrant varying degrees of coverage proportional to the assessed risk. With this idea, the potential budget impact might be reduced to roughly 20–30 million Baht (US$ 0.9–1.0 million) rather than 46.8 million Baht (US$ 1.5 million) as suggested above and the vaccination programme was still able to remain its cost effectiveness. Thus, for the next step of policy implementation, there should be a series of consultative meetings with a wide range of stakeholders to agree on the risk criteria and prioritisation process. Prisons that were prioritised as high risk should be paid greater attention than the others. All of these processes will also ensure that the implementation of the vaccination policy for prisoners is firmly grounded on evidence.

### 4.2. Methodological Discussion

To the authors’ knowledge, this study is likely to be the first study in Thailand, and possibly in Southeast Asia, that has performed a comprehensive analysis on a vaccination policy for prisoners, in terms of both health outcomes and budget impact. Despite a thorough analysis, this study still encountered some limitations. First, the vaccine efficacy varied year by year. It is possible that the recommended viral strains used for constituting the vaccine may not match the actual viral strains found in the outbreak. If this situation happens, the vaccination programme will be less cost effective. Second, many parameters were derived from model calibration and literature review rather than empirical data collection. These points also lead to a couple of recommendations: (i) further studies that account for uncertainty of parameters are needed (stochastic model), and (ii) if the vaccination policy is implemented, a monitoring system should be established in parallel. With a sound monitoring system in place, the policy will allow for the collection of feedback with empirical data from the field and these data will be useful to fine-tune the policy in subsequent years. Third, not all cost items in this study were an ‘economic cost’, some were literally a billing expense, which varied year by year due to the market price. Additional cost studies on outbreak investigation will be definitely helpful in terms of either academic angle or policy angle. Fourth, in reality, it is possible that the outbreaks in a prison originate from people outside the prison, for instance, visitors, wardens, transporters, and police. In other words, the movement of people entering and leaving the prisons is likely to be far more dynamic than the assumption used for performing the above sensitivity analysis. Hence, a more thorough analysis on the vaccination programme necessitates further information from this group of people. This includes data about the contact time, frequency of contact, and existing health status of people who come into contact with prisoners. However, the data on this population are not systematically recoded at the status quo. Fifth, the contact pattern between a case and a susceptible person may not occur at random, as assumed in this study. Again, to shed light on this point, an empirical data collection is of great value. Last but not least, this study did not deliberately account for the feasibility of the policy. There were many unobserved variables that have not been included in the model; for example, the number of health personnel to administer vaccines, the capacity of each prison to manage the vaccination, and the capacity of the outbreak investigation team (which might be overstretched if more than one outbreak occurs at the same time). As a result, the interpretation of the results should be made with great caution.

## 5. Conclusions

In conclusion, the vaccination programme was a cost-effective measure to halt influenza outbreaks amongst prisoners in Thailand. It could help reduce both the number of infected cases and all related costs, including the treatment cost and disease control cost. Rigorous surveillance systems and infection control practices should be set up to complement the vaccination policy. If a vaccination policy is to be implemented, a monitoring system should be established in tandem. Nevertheless, the more prisoners are vaccinated, the more the budget burden increases. Thus, the extent to which the prisoners are vaccinated hugely depends on the budget capacity of the Thai-DDC. Risk prioritisation on all prisons nationwide should be established. The Thai-DDC may consider implementing a full coverage programme in high-risk prisons, and the rest of the prisons should receive coverage that at least reaches the herd immunity threshold. Further empirical studies that aim to assess and monitor the actual impact of the programme are recommended.

## Figures and Tables

**Table 1:** Incidence of influenza outbreaks in prisons in Thailand, 2017–2019.

Year	Affected Province, n	Events, n	All Patients, n	Median Cases across Events, n (min-max)	Deaths, n	Dominant Subtype
2017	17	19	3052	105 (24–484)	2	Flu A, H1N1
2018	28	33	4756	120 (28–444)	1	Flu A, H1N1
2019 *	23	28	4481	81 (8–1204)	1	Flu B

Source: Division of Epidemiology (2019) [12]. Note: * Data in 2019 were recorded till May 2019.

**Table 2:** Key parameter list.

Parameter	Unit	Value	Reference
Total prisoners (whole country)	Persons	375,763	Department of Corrections, Ministry of Justice [18]
Total prisons	Prisons	143	Department of Corrections, Ministry of Justice [18]
Total prisoners per prison	Persons/prison	2628	Department of Corrections, Ministry of Justice [18]
Reproductive number	Dimensionless	1.40	Plans-Rubió [19] and model calibration
Prevalence of influenza	Dimensionless	0.002	Internal database of Department of Disease Control, Ministry of Public Health
Detection rate	Dimensionless	0.45	Karnjanapiboonwong et al. [20] and model calibration
Probability of turning from mild infection to severe infection	Dimensionless	0.01	Karnjanapiboonwong et al. [20]
Probability of death amongst severe cases	Dimensionless	0.01	Model calibration
Vaccine efficiency in preventing the turn from susceptible cases to mild cases	Dimensionless	0.36	Meeyai et al. [21]
Vaccine efficiency in preventing the turn from mild cases to severe cases	Dimensionless	0.67	Arriola et al. [22]
Vaccine efficiency in preventing the turn from severe cases to death cases	Dimensionless	0.66	Thompson et al. [16]
Duration of infection—turning from mild cases to recovered cases (no vaccination)	Days	5	Deiss et al. [23]
Duration of infection—turning from mild cases to recovered cases (vaccination)	Days	5	Deiss et al. [23]
Duration of turning from severe cases to recovered cases (no vaccination)	Days	7	Arriola et al. [22]
Duration of turning from severe cases to recovered cases (vaccination)	Days	7	Arriola et al. [22]
Duration of turning from mild cases to severe cases (no vaccination)	Days	2	Model calibration and internal database of Department of Disease Control, Ministry of Public Health
Duration of turning from mild cases to severe cases (vaccination)	Days	2	Model calibration and internal database of Department of Disease Control, Ministry of Public Health calibration
Efficacy of Oseltamivir in shortening clinical course from mild infection to recovery	Dimensionless	0.25	Fry et al. [24] and Lee et al. [25]
Efficacy of Oseltamivir in shortening clinical course from severe infection to recovery	Dimensionless	0.33	Fry et al. [24] and Lee et al. [25]
Efficacy of Oseltamivir in reducing the probability of turning from severe infection to death	Dimensionless	0.29	Fry et al. [24] and Lee et al. [25]
Efficacy of Oseltamivir in reducing the probability of turning from mild infection to severe infection	Dimensionless	0.46	Fry et al. [24] and Lee et al. [25]
Efficacy of outbreak control in breaking disease transmission by rapid response team	Dimensionless	0.80	Model calibration
Vaccination cost per person	Baht	97.50	Internal database of Department of Disease Control, Ministry of Public Health
Other logistic cost for vaccination per person	Baht	99.12	Meeyai et al. [21]
Laboratory cost per each round of outbreak investigation	Baht	25,000	Internal database of Department of Disease Control, Ministry of Public Health
Oseltamivir cost per person	Baht	250	Internal database of Department of Disease Control, Ministry of Public Health
Treatment cost per a mild case	Baht	432.41	Meeyai et al. [21]
Treatment cost per a severe case	Baht	15,723.23	Meeyai et al. [21]
Other logistic cost for outbreak investigation	Baht	20,000	Internal database of Department of Disease Control, Ministry of Public Health
DALYs per death	Years	9	Meeyai et al. [26]
DALYs per severely infected case	Years	0.022	Lugnér et al. [27]
DALYs per mildly infected case	Years	0.008	Lugnér et al. [27]

**Table 3:** Key formula used in the model.

Change of Status	Formula	Note
From susceptible cases to mildly infected cases (no vaccination)	dS/dt = − (SI_1_/N) × (R0/d_1_) × (1−f)	S = number of susceptible cases, I_1_ = number of mildly infected case, N = number of prisoners in a prison, R0 = basic reproductive number, d = duration of infection, f = effectiveness of outbreak investigation and control
From susceptible cases to mildly infected cases (vaccination)	dS/dt = − (SI_1_/N) × (R0/d_1_) × (1−f) × (1−v_1_)	S = number of susceptible cases, I_1_ = number of mildly infected case, N = number of prisoners in a prison, R0 = basic reproductive number, d = duration of infection, f = effectiveness of outbreak investigation and control, v_1_ = vaccine efficacy in reducing the probability of turning from susceptible case to mildly infected case
From mildly infected cases to severely infected cases (no vaccination)	dI_1_/dt = − (I_1_/d_2_) × p_1_ × (1−o_1_)	I_1_ = number of mildly infected case, d_2_ = duration of turning from mildly infected case to severely infected case, p_1_ = probability of turning from mildly infected case to severely infected case, o_1_ = efficacy of Oseltamivir in reducing the probability of mild infection to severe infection
From mildly infected cases to severely infected cases (vaccination)	dI_1_/dt = − (I_1_/d_2_) × p_1_ × (1−o_1_) × (1−v_2_)	I_1_ = number of mildly infected case, d_2_ = duration of turning from mildly infected case to severely infected case, p_1_ = probability of turning from mildly infected case to severely infected case, o_1_ = efficacy of Oseltamivir in reducing the probability of mild infection to severe infection, v_2_ = vaccine efficacy in reducing the probability of mild infection to severe infection
From mildly infected cases to recovered cases (no vaccination)	dI_1_/dt = − (I_1_/(d_3_ × (1−o_2_))) × (1−(p_1_ × (1−o_1_)))	I_1_ = number of mildly infected case, d_3_ = duration of turning from mildly infected case to recovered case, p_1_ = probability of turning from mildly infected case to recovered case, o_1_ = efficacy of Oseltamivir in reducing the probability of mild infection to severe infection, o_2_ = efficacy of Oseltamivir in shortening clinical course in mild case
From mildly infected cases to recovered cases (vaccination)	dI_1_/dt = − (I_1_/(d_3_ × (1−o_2_))) × (1−(p_1_ × (1−o_1_) × (1−v_2_)))	I_1_ = number of mildly infected case, d_3_ = duration of turning from mildly infected case to recovered case, p_1_ = probability of turning from mildly infected case to recovered case, o_1_ = efficacy of Oseltamivir in reducing the probability of mild infection to severe infection, o_2_ = efficacy of Oseltamivir in shortening clinical course in mild case, v_2_ = vaccine efficacy in reducing the probability of turning from mild infection to severe infection
From severely infected cases to death (no vaccination)	dI_2_/dt = − (I_2_/d_4_) × p_2_ × (1−o_3_)	I_2_ = number of severely infected case, d_4_ = duration of turning from severely infected case to death, o_3_ = efficacy of Oseltamivir in reducing the probability of death, p_2_ = probability of turning from severely infected case to death
From severely infected cases to death (vaccination)	dI_2_/dt = − (I_2_/d_4_) × p_2_ × (1−o_3_) × (1−v_3_)	I_2_ = number of severely infected case, d_4_ = duration of turning from severely infected case to death, o_3_ = efficacy of Oseltamivir in reducing the probability of death, p_2_ = probability of turning from severely infected case to death, v_3_ = vaccine efficacy in reducing the probability of turning severe infection to death
From severely infected cases to recovered cases (no vaccination)	dI_2_/dt = − (I_2_/(d_5_ × (1−o_4_))) × (1−(p_2_ × (1−o_3_)))	I_2_ = number of severely infected case, d_5_ = duration of turning from severe infection to recovery, o_3_ = efficacy of Oseltamivir in reducing the probability of death, o_4_ = efficacy of Oseltamivir in shortening clinical course in severe case, p_2_ = probability of turning from severely infected case to death
From severely infected cases to recovered (vaccination)	dI_2_/dt = − (I_2_/(d_5_ × (1−o_4_))) × (1−(p_2_ × (1−o_3_) × (1−v_3_)))	I_2_ = number of severely infected case, d_5_ = duration of turning from severe infection to recovery, o_3_ = efficacy of Oseltamivir in reducing the probability of death, o_4_ = efficacy of Oseltamivir in shortening clinical course in severe case, p_2_ = probability of turning from severely infected case to death, v_3_ = vaccine efficacy in reducing the probability of turning severe infection to death

**Table 4:** Scenarios of interest.

Scenario Code	Description
Routine^*^	Routine outbreak investigation
Routinevac10	Routine outbreak investigation plus 10% vaccination coverage
Routinevac30	Routine outbreak investigation plus 30% vaccination coverage
Routinevac100	Routine outbreak investigation plus 100% vaccination coverage
Extra	Doubling sensitivity of outbreak investigation
Extravac10	Doubling sensitivity of outbreak investigation plus 10% vaccination coverage
Extravac30	Doubling sensitivity of outbreak investigation plus 30% vaccination coverage
Extravac100	Doubling sensitivity of outbreak investigation plus 100% vaccination coverage

Note: * Reference scenario for ICER analysis.

**Table 5:** Overall results in each interested scenario.

Output	Routine	Routinevac10	Routinevac30	Routinevac100	Extra	Extravac10	Extravac30	Extravac100
Disease control related cost (million Baht)	1.3	7.0	19.1	46.8	10.0	14.0	21.3	46.8
• Vaccination cost	0.0	7.0	19.1	46.8	0.0	7.0	19.1	46.8
• Other outbreak investigation cost	1.3	0.0	0.0	0.0	10.0	6.9	2.2	0.0
Treatment related cost (million Baht)	173.5	162.3	128.3	44.1	148.9	138.5	119.7	44.1
• Treatment cost for mild cases	76.8	72.4	58.8	21.7	67.1	62.9	55.2	21.7
• Treatment cost for severe cases	96.7	89.9	69.5	22.4	81.7	75.6	64.5	22.4
Total volume of infected cases	185,398	173,184	140,390	51,703	160,367	150,213	131,732	51,703
Grand cost (million Baht)	174.8	169.3	147.5	90.9	158.9	152.4	141.1	90.9
Grand DALYs (years)	1583.4	1491.0	1203.6	438.4	1378.8	1289.9	1128.4	438.4

**Table 6:** ICER of all scenarios (focusing on disease control cost).

Comparison	Δ Cost (Million Baht)	DALY Averted (Years)	ICER (Baht per DALY Averted)
Routine *versus* Routinevac10	5.7	92.4	61,688.3
Routine *versus* Routinevac30	17.8	379.8	46,866.8
Routine *versus* Routinevac100	45.5	1145	39,738.0
Routine *versus* Extra	8.7	204.6	42,522.0
Routine *versus* Extravac10	12.7	293.5	43,270.9
Routine *versus* Extravac30	20.0	455	43,956.0
Routine *versus* Extravac100	45.5	1145	39,738.0

## Supplementary Materials

The following are available online at https://www.mdpi.com/1660-4601/17/4/1247/s1, Figure S1: Person model and Cost model.
