# Supplementary figures and images for: Cost Effectiveness and Budget Impact Analyses of Influenza Vaccination for Prisoners in Thailand: An Application of System Dynamic Modelling
Source: Int J Environ Res Public Health. 2020 Feb 14;17(4):1247. doi: 10.3390/ijerph17041247 (PMC7068611; doi:10.3390/ijerph17041247)

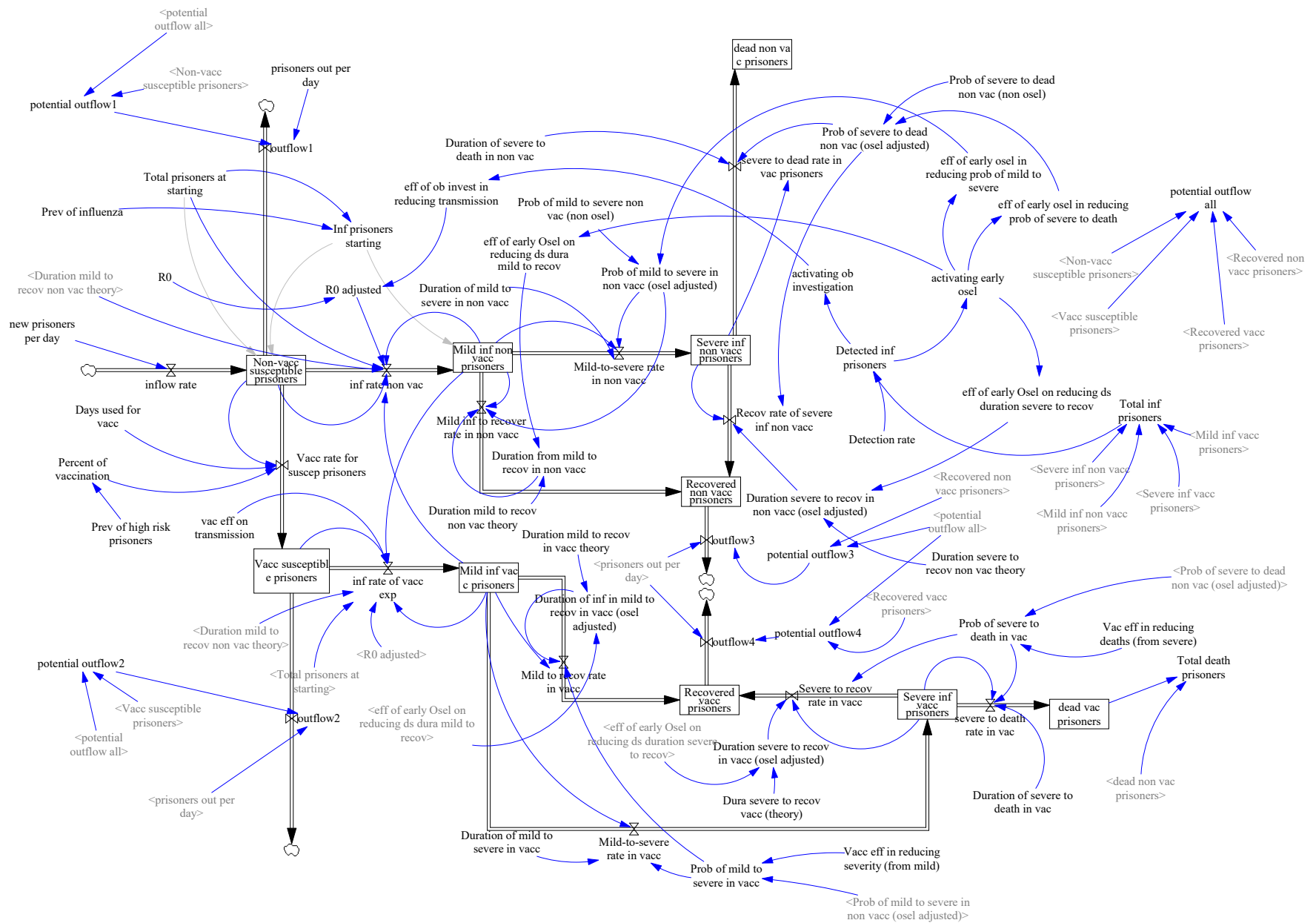

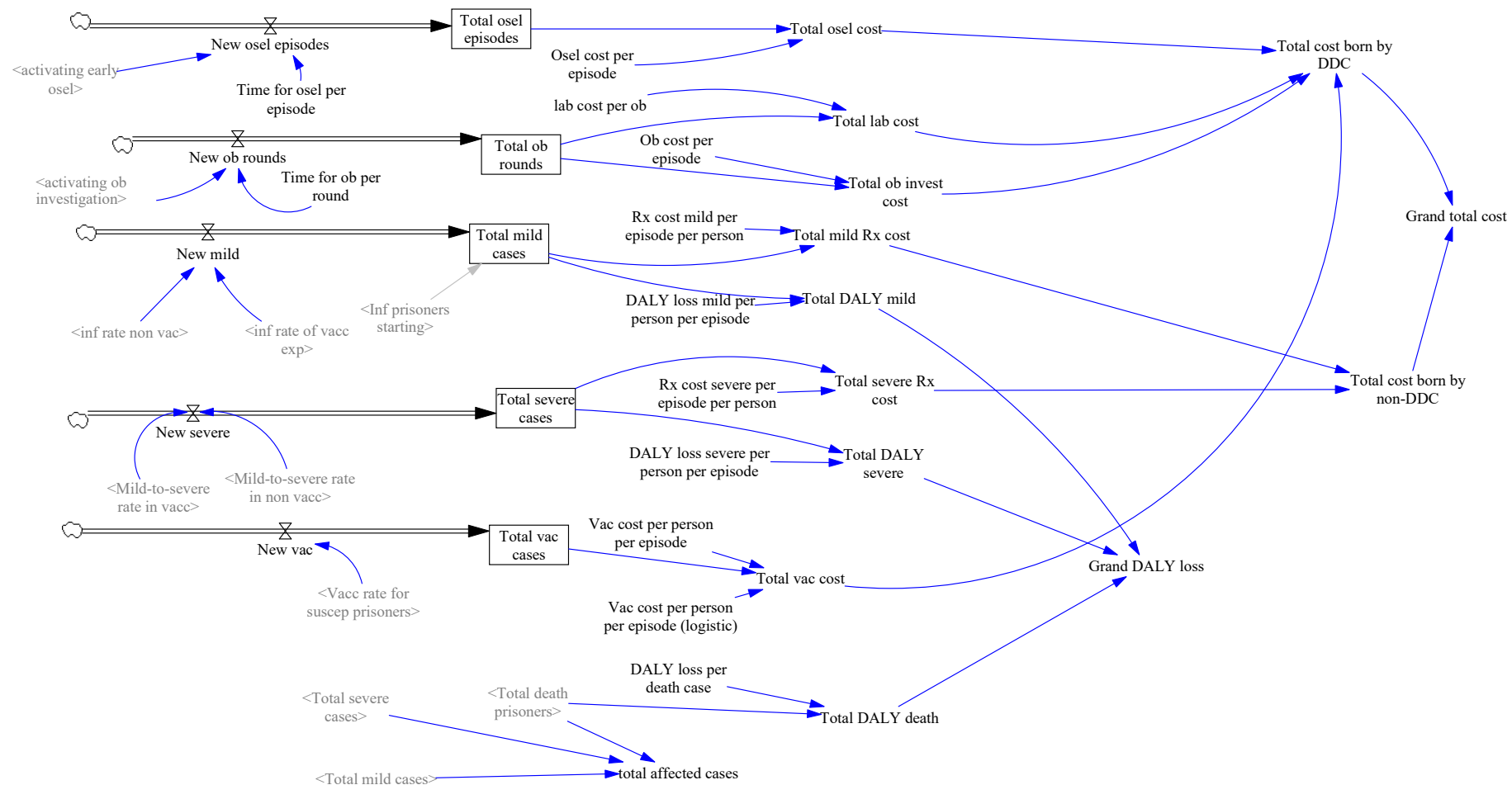

Supplement: Supplementary file 1 [file ijerph-17-01247-s001.pdf]
